# Urgent-Start Peritoneal Dialysis and Hemodialysis in ESRD Patients: Complications and Outcomes

**DOI:** 10.1371/journal.pone.0166181

**Published:** 2016-11-08

**Authors:** Haijiao Jin, Wei Fang, Mingli Zhu, Zanzhe Yu, Yan Fang, Hao Yan, Minfang Zhang, Qin Wang, Xiajing Che, Yuanyuan Xie, Jiaying Huang, Chunhua Hu, Haifen Zhang, Shan Mou, Zhaohui Ni

**Affiliations:** Department of Nephrology, Renji Hospital, School of Medicine, Shanghai Jiao Tong University, Shanghai, China; Hospital Universitario de la Princesa, SPAIN

## Abstract

**Background:**

Several studies have suggested that urgent-start peritoneal dialysis (PD) is a feasible alternative to hemodialysis (HD) in patients with end-stage renal disease (ESRD), but the impact of the dialysis modality on outcome, especially on short-term complications, in urgent-start dialysis has not been directly evaluated. The aim of the current study was to compare the complications and outcomes of PD and HD in urgent-start dialysis ESRD patients.

**Methods:**

In this retrospective study, ESRD patients who initiated dialysis urgently without a pre-established functional vascular access or PD catheter at a single center from January 2013 to December 2014 were included. Patients were grouped according to their dialysis modality (PD and HD). Each patient was followed for at least 30 days after catheter insertion (until January 2016). Dialysis-related complications and patient survival were compared between the two groups.

**Results:**

Our study enrolled 178 patients (56.2% male), of whom 96 and 82 patients were in the PD and HD groups, respectively. Compared with HD patients, PD patients had more cardiovascular disease, less heart failure, higher levels of serum potassium, hemoglobin, serum albumin, serum pre-albumin, and lower levels of brain natriuretic peptide. There were no significant differences in gender, age, use of steroids, early referral to a nephrologist, prevalence of primary renal diseases, prevalence of co-morbidities, and other laboratory characteristics between the groups. The incidence of dialysis-related complications during the first 30 days was significantly higher in HD than PD patients. HD patients had a significantly higher probability of bacteremia compared to PD patients. HD was an independent predictor of short-term (30-day) dialysis-related complications. There was no significant difference between PD and HD patients with respect to patient survival rate.

**Conclusion:**

In an experienced center, PD is a safe and feasible dialysis alternative to HD for ESRD patients with an urgent need for dialysis.

## Introduction

The prevalence of chronic kidney disease (CKD) and end-stage renal disease (ESRD) is on the rise worldwide. According to the United States Renal Data System (USRDS) 2014 annual data report, the prevalence of CKD and ESRD was 13.6% and 0.14%, respectively [1, 2]. Moreover, many patients who progress to ESRD, even with regular nephrology follow-up, do not have a distinct plan at the time of initiating dialysis therapy, resulting in an urgent need for dialysis. Urgent-start dialysis refers to urgent initiation of dialysis for ESRD patients with no pre-established functional vascular access or peritoneal dialysis (PD) catheter. Hemodialysis (HD) is preferred in most centers with a high rate of central venous catheter (CVC) use at the time of initiating dialysis among HD patients [3]. There is a significantly increased risk of infectious complications, thrombosis, and other complications associated with CVC use which negatively affects patient prognosis [4]. Within the last decade, urgent-start PD has gained considerable interest amongst nephrologists. Several publications have provided assurances that urgent-start PD is indeed feasible and can serve patients well [5–11]; however, most of the studies have small sample sizes, and the impact of the urgent-start dialysis modality on outcome, especially on short-term complications, has not been directly evaluated. Therefore, we compared the dialysis-related complications and survival rate directly between urgent-start PD and HD groups with a large sample to determine the feasibility and safety of urgent-start PD as an alternate initial modality of dialysis for patients who require urgent initiation of dialysis therapy.

## Patients and Methods

Our research was approved by the Institutional Review Board of Ren Ji Hospital, School of Medicine, Shanghai Jiao Tong University. Written consents were obtained from the participants. All ESRD patients, 18–85 years of age, who urgently initiated dialysis therapy at Renji Hospital of Shanghai Jiao Tong University School of Medicine between 1 January 2013 and 31 December 2014 were included in the study. Urgent-start dialysis was defined as ESRD patients who required urgent initiation of dialysis without pre-established functional vascular access or a PD catheter. Exclusion criteria, which was used to exclude those patients who were not able to tolerate PD catheter insertion or wait time for PD procedure, included severe respiratory insufficiency, severe acute heart failure, severe hyperkalemia (>6.5 mmol/L), and severe acidosis (serum bicarbonate <12 mmol/L). Seventy-nine patients were excluded. All of the patients received a CVC immediately and started HD immediately after catheter insertion. All patients included in this study were educated about renal replacement therapy modalities (both PD and HD). The patients were provided a modality recommendation by an experienced nephrologist, but freely made their own decision regarding the dialysis modality. Patients were grouped according to the dialysis modality (PD and HD). Decisions of when to start dialysis therapy were made by experienced nephrologists on the basis of clinical conditions and laboratory parameters of individual patients. Each patient was followed for at least 30 days after catheter insertion (until January 2016). The study was conducted between March 2016 and June 2016. We had access to information that identified individual participants during or after data collection. Logistic regression analysis and Kaplan–Meier curves were used to compare complications and outcomes in two groups.

In PD patients, all Tenckhoff catheter insertions were performed by experienced nephrologists using the laparotomy method [12] and adhering to one protocol. The key points of the procedure for catheter insertion included bowel preparation during the peri-operative period, administration of prophylactic antibiotics (intravenous cephalosporin or vancomycin) at the time of catheter insertion, placement of the catheter in a downward direction with the superficial cuff 2–3 cm from the exit site, and testing of catheter function by filling and draining PD fluid before tunneling. The time from placement-to-PD was determined by the nephrologists based on the clinical condition of the individual patient within 14 days. A low intraperitoneal volume (0.75–1.2 L) in the supine position was used to avoid leakages, which was gradually increased to 2 L per exchange within 2 weeks after catheter insertion in continuous ambulatory peritoneal dialysis (CAPD), daytime ambulatory peritoneal dialysis (DAPD), intermittent peritoneal dialysis (IPD), or automated peritoneal dialysis (APD). For CAPD, DAPD, and IPD patients, two or four exchanges were performed daily; for APD patients, six or seven cycles were performed daily. All patients were dialyzed using a glucose-based PD solution (Dianeal; Baxter China, Shanghai, PR China).

All CVCs were inserted in HD patients under local anesthesia by experienced nephrologists following the Seldinger technique [13] in the internal jugular or femoral vein. HD patients were treated by HD for 6–12 h/week (blood flow, 180–200 mL/min; dialysate flow, 500 mL/min; ultrafiltration, 0.4–0.5 L) or by continuous renal replacement therapy (RRT) for 6–24 h/week (replacement fluid flow, 2000 mL/h; dialysate flow, 2000 mL/h; ultrafiltration, 0.4–0.5 L). The choice of dialysis prescription was made on the basis of the clinical status of each individual patient. All CVCs were double-lumen 11.5-F catheters (Mahurkar; Kendall-Tyco Healthcare, Shanghai, China).

## Data Collection

The data collected included patient demographics, primary diseases, co-morbid diseases, medical history, and laboratory parameters. Data recorded at the time of initiating dialysis included age, gender, primary etiology of ESRD, presence of co-morbid diseases (diabetes, hypertension, cardiovascular disease, chronic heart failure [New York Heart Association {NYHA} stage III-IV], cerebrovascular disease, and malignancy, Charlson co-morbidity index [CCI]), use of steroids, and early referral to nephrologists in 6 months. Laboratory parameters were collected at the time of initiating dialysis, including the estimated glomerular filtration rate, serum creatinine, serum urea, serum sodium, serum potassium, pH, serum bicarbonate, brain natriuretic peptide, serum albumin, serum pre-albumin, hemoglobin, serum calcium, serum corrected calcium, serum phosphate, parathyroid hormone, triglycerides, total cholesterol, low-density lipoprotein, and high-density lipoprotein.

The primary outcomes of the study were the incidence of dialysis-related complications (infectious and non-infectious complications) and dialysis-related complications requiring catheter re-insertion and bacteremia during the first 30 days after catheter insertion. Dialysis-related complications (episodes, type, intervention strategy, and outcome) and patient outcomes (death, transfer to other centers, or loss to follow-up) were carefully tracked and recorded.

## Statistical Analysis

All results are expressed as the mean ± standard deviation for normally distributed data and frequencies and percentages for non-normally distributed and categorical data. Differences between the groups in patient demographics and clinical and laboratory parameters were evaluated by t-test for normally distributed data or Mann–Whitney non-parametric test for non-normally distributed data. Comparisons of percentages between groups were performed using the chi-square test. Logistic regression analysis was used to determine the factors associated with dialysis-related complications and patient survival rate. Variables with a p value <0.10 based on univariate analysis were introduced in logistic regression analysis using the backward elimination method. Kaplan–Meier curves and log-rank test were used to compare the patient survival rate between groups.

All statistical analyses were performed using SPSS for Windows (version 19.0; SPSS, Inc., Chicago, IL, USA). A p value <0.05 was considered statistically significant.

## Results

### Demographic and clinical characteristics

**Fig 1:**
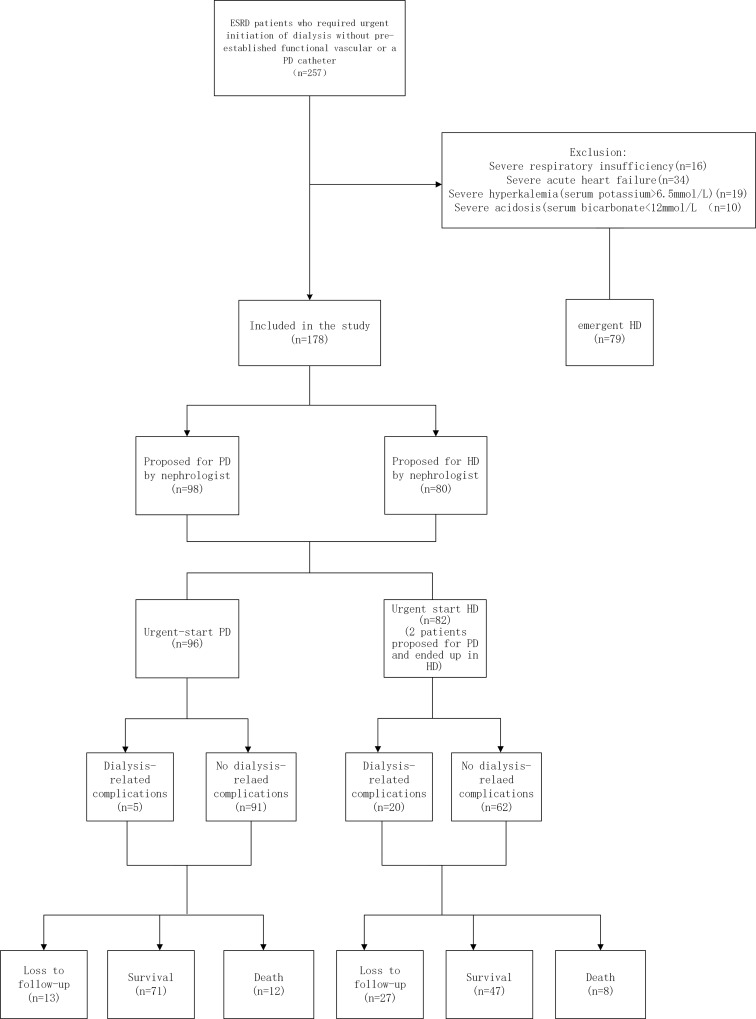
Patient flowchart.

Fig 1 presents the patient flow procedure. Our study enrolled 178 patients (56.2% male), including 96 (53.9%) PD patients and 82 (46.1%) HD patients. The mean age was 53.4±19.0 years (range, 18–85 years). In the PD group, patients received a catheter during the first 1–4 days after referral to a nephrologist and initiated dialysis during the first 1–10 days after insertion. The median break-in period (from catheter insertion-to-dialysis initiation) was 4 days. In the HD group, patients received a catheter during the first 1–2 days after referral to a nephrologist and started dialysis on the same day or the day after catheter insertion.

Compared with HD patients, PD patients had more cardiovascular disease, less heart failure, higher levels of serum potassium, hemoglobin, serum albumin, and serum pre-albumin, and lower levels of brain natriuretic peptide. There were no significant differences in terms of gender, age, use of steroids, early referral to a nephrologist in the past 6 months, prevalence of primary renal diseases, prevalence of co-morbidities (diabetes mellitus, hypertension, cerebrovascular diseases, and malignancies), CCI, and other clinical characteristics between the groups (all p >0.05). Tables 1 and 2 show the baseline demographic and clinical characteristics of the study patients.

**Table 1:** Demographic and Baseline Characteristics of the Study Patients.

Characteristics	Overall (n = 178)	PD (n = 96)	HD (n = 82)	p Value
**Gender [n (%) men]**	100 (56.2%)	56 (58.3%)	44 (53.7%)	0.531
**Mean age (years)**	53.4±19.0	55.2±17.9	51.2±20.0	0.172
**Primary diseases [n (%)]**				
**Chronic glomerulonephritis**	78 (43.8%)	36 (37.5%)	42 (51.2%)	0.066
**Diabetic nephropathy**	35 (19.7%)	21 (21.9%)	14 (17.1%)	0.422
**Hypertensive nephrosclerosis**	9 (5.1%)	5 (5.2%)	4 (4.9%)	1.000
**Polycystic kidney disease**	7 (4.0%)	4 (4.2%)	3 (3.7%)	1.000
**Vasculitis nephritis**	3 (1.7%)	2 (2.1%)	1 (1.2%)	1.000
**Lupus nephritis**	7 (4.0%)	5 (5.2%)	2 (2.4%)	0.575
**Gouty nephropathy**	3 (1.7%)	2 (2.1%)	1 (1.2%)	1.000
**Unknown**	36 (20.2%)	21 (21.9%)	15 (18.3%)	0.501
**Steroid use [n (%)]**	29 (16.3%)	11 (11.5%)	18 (22.0%)	0.059
**Early referral to nephrologist in 6 months [n (%)]**	99 (55.6%)	52 (54.2%)	47 (57.3%)	0.673
**Co-morbidities [n (%)]**				
**Diabetes**	51 (28.7%)	31 (32.3%)	20 (24.4%)	0.245
**Hypertension**	130 (73.0%)	70 (72.9%)	60 (73.2%)	0.970
**Cardiac vascular diseases**	29 (16.3%)	21 (21.9%)	8 (9.8%)	0.029
**Heart failure**	66 (37.1%)	26 (30.2%)	40 (48.8%)	0.003
**Cerebrovascular disease**	16 (9.0%)	8 (8.3%)	8 (9.8%)	0.741
**Malignancy**	7 (4.0%)	2 (2.1%)	5 (6.1%)	0.324
**Charlson co-morbidity index (CCI)**	3.48±1.51	3.47±1.58	3.50±1.43	0.891

**Table 2:** Baseline Laboratory Characteristics of the Study Patients.

Characteristics	Overall (n = 178)	PD (n = 96)	HD (n = 82)	p Value
**eGFR (mL/min/1.73 m**^**2**^**)**	5.428 (4.4045,6.906)	5.354 (4.425,6.311)	5.620 (3.607,7. 973)	0.747
**Mean serum creatinine (umol/L)**	846.4 (673.8, 998.7)	828.0 (619.9, 947.8)	868.3 (618.7, 1121.3)	0.576
**Mean serum urea (mmol/L)**	29.8±10.1	28.9±8.1	30.8±11.9	0.227
**Mean serum sodium (mmol/L)**	138.0±4.9	138.5±4.7	137.3±5.2	0.093
**Mean serum potassium (mmol/L)**	4.4±0.8	4.5±0.8	4.3±0.8	0.038
**pH**	7.34 (7.31, 7.38)	7.34 (7.31, 7.37)	7.35 (7.31, 7.40)	0.076
**Mean bicarbonate (mmol/L)**	21.2±4.2	21.5±4.0	20.8±4.4	0.253
**BNP (pg/mL)**	409.0 (155.0, 934.5)	328.5 (129.5, 776.8)	503.5 (206.0, 1430.0)	0.008
**Mean serum albumin (g/L)**	32.5±6.3	33.5±5.7	31.3±6.7	0.022
**Mean serum pre-albumin (mg/L)**	282.7±85.0	304.5±78.0	257.0±86.1	<0.001
**Hemoglobin (g/L)**	78.6±20.2	81.5±17.7	75.3±22.5	0.039
**Mean serum calcium (mmol/L)**	1.91 (1.74, 2.06)	1.95 (1.74, 2.07)	1.88 (1.74, 2.06)	0.663
**Mean corrected calcium (mmol/L)**	2.09 (1.89, 2.23)	2.08 (1.85, 2.21)	2.12 (1.92, 2.25)	0.336
**Mean phosphate (mmol/L)**	2.04 (1.67, 2.48)	2.08 (1.76, 2.47)	1.92 (1.61, 2.49)	0.300
**PTH (ng/L)**	303.5 (165.8, 481.0)	316.0 (202.0, 479.5)	300.0 (130.0, 481.8)	0.330

There were no differences in TG, TC, HDL, and LDL concentrations. eGFR, estimated glomerular filtration rate; BNP, brain natriuretic peptide; PTH, parathyroid hormone; TG, triglycerides; TC, total cholesterol; HDL, high-density lipoprotein; LDL, low-density lipoprotein.

### Dialysis-related complications

During the first 30 days after catheter insertion, 5 PD patients (5.2%) and 20 HD patients (24.4%) developed dialysis-related complications (Table 3). Three PD patients developed non-infectious complications (all malpositioned), but only one required surgical intervention. No patient developed severe or life-threatening complications, such as severe bleeding, leakage, and organ rupture. Two PD patients developed infectious complications (both had peritonitis), and both patients were cured after standard treatment. Among the 20 HD patients who developed dialysis-related complications, all required catheter re-insertion. Eleven HD patients developed non-infectious complications, including bleeding (n = 3), thrombosis (n = 6), and self-removal (n = 2). Logistic regression analysis showed that urgent-start HD was an independent risk factor in patients with short-term dialysis-related complications (Table 4).

**Table 3:** Dialysis-related Complications in the Study Groups during a 30-day Period.

Complication	Overall (n = 178)	PD (n = 96)	HD (n = 82)	p Value
**Dialysis-related complications [n (%)]**	25 (14.0%)	5 (5.2%)	20 (24.4%)	<0.001
**Dialysis-related complications requiring re-insertion [n (%)]**	21 (11.8%)	1 (1.0%)	20 (24.4%)	<0.001
**Non-infectious complications [n (%)]**	14 (7.0%)	3 (3.1%)	11 (13.4%)	0.011
**Bleeding [n (%)]**	3 (1.7%)	0 (0%)	3 (3.7%)	0.192
**Leakage [n (%)]**	0 (0%)	0 (0%)	0 (0%)	/
**Organ rupture [n (%)]**	0 (0%)	0 (0%)	0 (0%)	/
**Thrombosis [n (%)]**	6 (3.4%)	0 (0%)	6 (7.3%)	0.023
**Self-remove [n (%)]**	2 (1.1%)	0 (0%)	2 (2.4%)	0.409
**Malposition [n(%)]**	3 (1.7%)	3 (3.1%)	0 (0%)	0.303
**Infectious complications [n (%)]**	11 (6.2%)	2 (2.1%)	9 (11.0%)	0.014
**Peritonitis [n (%)]**	2 (1.1%)	2 (2.1%)	0 (0%)	0.548
**Catheter-related infection [n (%)]**	9 (5.1%)	0 (0%)	9 (11.0%)	0.003

**Table 4:** Predictors of Short-term Dialysis-related Complications by Logistic Regression Analysis.

Factor	OR	95% CI	p Value
**Urgent-start HD versus PD**	5.024	1.760–14.341	0.003
**Heart failure (NYHA III-IV)**	2.261	0.915–5.585	0.077

### Bacteremia

Three PD patients (3.1%) and 11 HD patients (13.4%) developed bacteremia within 30 days after catheter insertion. HD patients had a significantly higher proportion of bacteremia in the first 30 days compared to PD patients (p = 0.011). Among the 11 HD patients who had bacteremia, 9 were related to catheter infections and the other 2 were related to severe pulmonary infections. Causes of bacteremia in the 3 PD patients included pulmonary infection (n = 2) and peritonitis (n = 1).

### Patient survival

**Fig 2:**
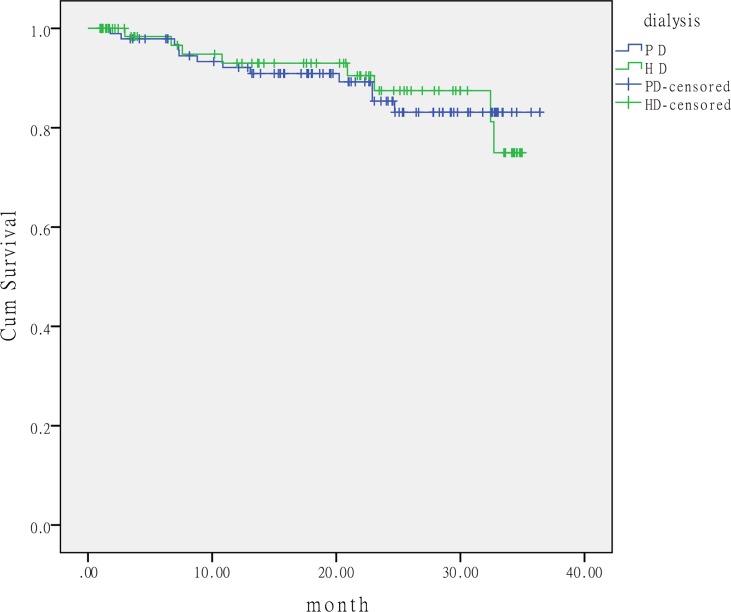
Patient survival curves for study groups. No difference between PD and HD groups in patient survival (log rank, 0.004; p = 0.947). PD: peritoneal dialysis; HD: hemodialysis.

In each group, the actuarial patient survival at 3 months was 97.9% for PD patients and 98.4% for HD patients. The actuarial patient survival at 1 year was 92.1% for PD patients and 93% for HD patients. Kaplan–Meier curves revealed no significant difference in patient survival between the two groups (Fig 2). Logistic regression analysis showed that low serum potassium and albumin levels were risk factors for patient survival, but the urgent-start dialysis modality was not correlated with patient survival (Table 5).

**Table 5:** Predictors of Patient Survival by Logistic Regression Analysis.

Factor	OR	95% CI	p Value
**Serum potassium (by 1 mmol/L increase)**	0.434	0.227–0.832	0.012
**Albumin (by 1 g/L increase)**	0.914	0.843–0.991	0.030

## Discussion

This is the first study with a large sample to directly compare short-term complications and patient survival in urgent-start PD and HD patients. Our results suggest that patients who started urgent-start PD experienced a lower risk of short-term dialysis-related complications, but did not correlate with patient survival. Urgent-start HD is an independent risk factor for short-term dialysis-related complications.

In 2010, 2.618 million people received RRT worldwide and at least 2.284 million people are thought to have died prematurely because RRT could not be accessed. In Asia, 1.907 million people need, but cannot receive RRT. The worldwide use of RRT is projected to more than double by 2030, with the most growth in Asia [14]. Except for several countries and regions, such as Mexico and Hong Kong, HD is still the dominant dialysis modality worldwide, with 70%–80% of patients initiating dialysis with HD [2]. The Chinese government attaches great importance to the treatment of ESRD patients. ESRD is covered by the basic healthcare insurance and new rural cooperative medical care systems. Moreover, in 2012 China announced a decision to expand the coverage of the country’s healthcare insurance system to embrace critical illness (including ESRD), thus aiming to prevent patients from being reduced to poverty by necessary healthcare costs.

According to the USRDS data report, 60% of patients who progress to ESRD do not have a distinct plan at the time of initiating dialysis therapy [15]. In our study, even though one-half of patients had a previous ESRD nephrology follow-up and were informed about dialysis techniques, early education and adequate preparation were not achieved because many patients did not seek timely care, even when aware. In addition, some stable patients had acute worsening kidney function that was not predictable, resulting in an urgent need for dialysis. CVC continues to be the most common urgent-start dialysis access, representing approximately 80% of all accesses [3]; however, multiple studies have demonstrated a variety of complications associated with the placement and use of CVC, including catheter-related infections, thromboses, catheter malfunction, and hemodynamic instability, resulting in a negative effect on patient survival [16–18]. Lee et al. [19] reported that the risk of CVC-related infection is close to 50% by 6 months. Infection due to CVC is associated with high morbidity and mortality [20, 21]. Therefore, it is important to limit CVC use to reduce the risk of catheter-related complications.

Early in its history, several limitations precluded the widespread use of PD for urgent-start dialysis, including a high incidence of leakage, improper position, and peritonitis. With technologic improvement of PD, such as design of the double-cuffed PD catheter, use of reverse osmosis for the production of sterile PD solutions, and the introduction of APD, many complications could be prevented. Although international guidelines recommend that catheter insertion should be performed at least 2 weeks before starting PD, small dialysate volumes in the supine position can be used if dialysis is required earlier [12, 22].

Recently, there has been mounting evidence on the feasibility of PD as an alternative to HD as an urgent-start dialysis modality [5–11]. These studies have concluded that PD is equivalent to HD with respect to patient survival, and an additional benefit of PD is fewer short-term dialysis-related complications. In addition, Liu et al. [23] characterized the costs associated with different urgent-start modalities over the first 90 days of treatment from a provider perspective. Liu et al. [23] reported the estimated per patient cost over the first 90 days for urgent-start PD and HD was $16,398 and $19,352, respectively. Thus, urgent-start PD is a cost-saving approach for the initiation of dialysis in patients requiring urgent-start dialysis [23].

Shame et al. [24] reported that after a tight catheter was secured during the insertion, the overall incidence of peri-catheter leakage remained low in the entire study cohort, and the incidence of peri-catheter leakage did not increase despite a shorter break-in period. Povlsen and Ivarsen [5] retrospectively described how acute APD was initiated using a standard prescription for a 12-h overnight APD in the supine position immediately after PD catheter placement and compared short-term outcome measures and dialysis-related complications between urgent-start and planned-start patients. Povlsen and Ivarsen [5] reported that there was no significant difference in the number and type of infectious complications between the two groups despite higher mechanical complications in the acute group compared with the planned group (p <0.05). There was no difference in short-term PD technique survival rates between the two groups (86.7% vs. 90.0%) [5]. A small sample size, prospective, randomized study reported that peritonitis, exit-site infections, catheter-related complications, and other complications were similar between the urgent-start PD and non-urgent-start groups, although the number of minor leaks was higher in the urgent-start PD group [6]. More recently, another small sample size, prospective study showed the safety and feasibility of urgent-start PD in a developing country [7]. Among 35 patients with urgent initiation of PD, peritonitis and mechanical complications occurred in 14.2% and 25.7%, respectively. Technique survival was 85.7% [7]. A previous large size, retrospective study (n = 657) in our center also showed that a break-in period of <1 week might result in a minor increased risk of mechanical complications, but might have no major effect on technique survival in PD patients [25]. In the current study, only five PD patients had dialysis-related complications in the first 30 days after catheter insertion and only one required surgical intervention. None of the complications were severe or life-threatening. Considering our center had extensive experience in PD catheter implantation and management, our findings showed that at least in an experienced center, PD could be an alternative dialysis modality for urgent-start dialysis ESRD patients.

There is limited evidence directly comparing dialysis-related complications between urgent-start PD and HD [8, 9]. Koch et al. [8] showed that unplanned HD patients had a significantly higher probability of bacteremia in the first 183 days compared to PD patients (21.1 vs. 3.0%, p <0.01), whereas the risk for peritonitis was not significantly different in the two groups (1.8% vs. 1.5%, p = 1.000). Consistent with the findings of Koch et al. [8] we found that the incidence of bacteremia was considerably higher in HD patients compared to PD patients.

Lobbedez et al. [9] reported that actuarial patient survival at 1 year was 79% for unplanned HD compared with 83% for unplanned PD. After adjustment of the initial modified CCI, dialysis modality had no impact on patient survival [9]. Koch et al. [8] also reported there was no significant difference in half-year mortality in unplanned PD patients versus unplanned HD patients (30.3% vs. 42.1%, p = 0.19). In the current study, the actuarial patient survival at 3 months and 1 year was 97.9% and 92.1% for PD patients, and 98.4% and 93% for HD patients, respectively. No significant difference in patient survival existed between the two groups. Our findings suggest that urgent-start PD did not have a negative effect on patient survival as an urgent dialysis modality.

Our study had several limitations. The study was a single-center, non-matched, retrospective study. The single center nature of the study also limited the generalizability of the results. The dialysis modality was recommended by individual nephrologists based on patient condition and ultimately determined by the patient. Decisions of when to start dialysis therapy were made by an experienced nephrologist on the basis of the clinical status and laboratory parameters of the individual patient. Although all of the nephrologists practicing PD were experienced physicians and would typically make the same decision, individual bias cannot be completely avoided. In addition, urgent-start HD patients were in more critical conditions in our study with lower levels of potassium, hemoglobin, serum albumin, and serum pre-albumin, and higher levels of brain natriuretic peptide. Moreover, logistic regression analysis showed that low serum potassium and albumin levels were risk factors for patient survival, which might result in worse outcomes. Thus, additional research should focus not only on what type of dialysis access should be used, but also on whom. Clearly, prospective randomized controlled trials are needed to definitively demonstrate the optimal dialysis prescription in urgent-start PD patients.

## Conclusion

Our study suggested that peritoneal dialysis is a safe and feasible alternative to hemodialysis for urgent dialysis in ESRD patients at an experienced center.

## Supporting Information

S1 File Data underlying the results. doi:10.5061/dryad.20h3k
(XLS)

S2 File STROBE checklist. (DOCX)
