# Supplementary material for: Urgent-Start Peritoneal Dialysis and Hemodialysis in ESRD Patients: Complications and Outcomes
Source: PLoS One. 2016 Nov 8;11(11):e0166181. doi: 10.1371/journal.pone.0166181 (PMC5100934; doi:10.1371/journal.pone.0166181)
Supplement: S2 File — (DOCX) [file pone.0166181.s002.docx]

STROBE Statement—checklist of items that should be included in reports of observational studies

|  | Item No. | Recommendation | Page  No. | Relevant text from manuscript |
| --- | --- | --- | --- | --- |
| **Title and abstract** | 1 | (*a*) Indicate the study’s design with a commonly used term in the title or the abstract | 1 | Urgent-start Peritoneal Dialysis and Hemodialysis in ESRD Patients: Complications and Outcomes |
|  |  | (*b*) Provide in the abstract an informative and balanced summary of what was done and what was found | 2 | **Background.** Several studies have suggested that urgent-start peritoneal dialysis (PD) is a feasible alternative to hemodialysis (HD) in patients with end-stage renal disease (ESRD), but the impact of the dialysis modality on outcome, especially on short-term complications, in urgent-start dialysis has not been directly evaluated. The aim of the current study was to compare the complications and outcomes of PD and HD in urgent-start dialysis ESRD patients.  **Methods.** In this retrospective study, ESRD patients who initiated dialysis urgently without a pre-established functional vascular access or PD catheter at a single center from January 2013 to December 2014 were included. Patients were grouped according to their dialysis modality (PD and HD). Each patient was followed for at least 30 days after catheter insertion (until January 2016). Dialysis-related complications and patient survival were compared between the two groups.  **Results.** Our study enrolled 178 patients (56.2% male), of whom 96 and 82 patients were in the PD and HD groups, respectively. Compared with HD patients, PD patients had more cardiovascular disease, less heart failure, higher levels of serum potassium, hemoglobin, serum albumin, serum pre-albumin, and lower levels of brain natriuretic peptide. There were no significant differences in gender, age, use of steroids, early referral to a nephrologist, prevalence of primary renal diseases, prevalence of co-morbidities, and other laboratory characteristics between the groups. The incidence of dialysis-related complications during the first 30 days was significantly higher in HD than PD patients. HD patients had a significantly higher probability of bacteremia compared to PD patients. HD was an independent predictor of short-term (30-day) dialysis-related complications. There was no significant difference between PD and HD patients with respect to patient survival rate.  **Conclusion.** In an experienced center, PD is a safe and feasible dialysis alternative to HD for ESRD patients with an urgent need for dialysis. |
| Introduction | | | |  |
| Background/rationale | 2 | Explain the scientific background and rationale for the investigation being reported | 3-4 | The prevalence of chronic kidney disease (CKD) and end-stage renal disease (ESRD) is on the rise worldwide. According to the United States Renal Data System (USRDS) 2014 annual data report, the prevalence of CKD and ESRD was 13.6% and 0.14%, respectively. Moreover, many patients who progress to ESRD, even with regular nephrology follow-up, do not have a distinct plan at the time of initiating dialysis therapy, resulting in an urgent need for dialysis. Urgent-start dialysis refers to urgent initiation of dialysis for ESRD patients with no pre-established functional vascular access or peritoneal dialysis (PD) catheter. Hemodialysis (HD) is preferred in most centers with a high rate of central venous catheter (CVC) use at the time of initiating dialysis among HD patients. There is a significantly increased risk of infectious complications, thrombosis, and other complications associated with CVC use which negatively affects patient prognosis. Within the last decade, urgent-start PD has gained considerable interest amongst nephrologists. Several publications have provided assurances that urgent-start PD is indeed feasible and can serve patients well; however, most of the studies have small sample sizes, and the impact of the urgent-start dialysis modality on outcome, especially on short-term complications, has not been directly evaluated. |
| Objectives | 3 | State specific objectives, including any prespecified hypotheses | 4 | We compared the dialysis-related complications and survival rate directly between urgent-start PD and HD groups with a large sample to determine the feasibility and safety of urgent-start PD as an alternate initial modality of dialysis for patients who require urgent initiation of dialysis therapy. |
| Methods | | | |  |
| Study design | 4 | Present key elements of study design early in the paper | 2 | In this retrospective study, ESRD patients who initiated dialysis urgently without a pre-established functional vascular access or PD catheter at a single center from January 2013 to December 2014 were included. Patients were grouped according to their dialysis modality (PD and HD). Each patient was followed for at least 30 days after catheter insertion (until January 2016). Dialysis-related complications and patient survival were compared between the two groups. |
| Setting | 5 | Describe the setting, locations, and relevant dates, including periods of recruitment, exposure, follow-up, and data collection | 4-6 | All ESRD patients, 18–85 years of age, who urgently initiated dialysis therapy at Renji Hospital of Shanghai Jiao Tong University School of Medicine between 1 January 2013 and 31 December 2014 were included in the study. Each patient was followed for at least 30 days after catheter insertion (until January 2016). The data collected included patient demographics, primary diseases, co-morbid diseases, medical history, and laboratory parameters. Data recorded at the time of initiating dialysis included age, gender, primary etiology of ESRD, presence of co-morbid diseases (diabetes, hypertension, cardiovascular disease, chronic heart failure [New York Heart Association {NYHA} stage III-IV], cerebrovascular disease, and malignancy, Charlson co-morbidity index [CCI]), use of steroids, and early referral to nephrologists in 6 months. Laboratory parameters were collected at the time of initiating dialysis, including the estimated glomerular filtration rate, serum creatinine, serum urea, serum sodium, serum potassium, pH, serum bicarbonate, brain natriuretic peptide, serum albumin, serum pre-albumin, hemoglobin, serum calcium, serum corrected calcium, serum phosphate, parathyroid hormone, triglycerides, total cholesterol, low-density lipoprotein, and high-density lipoprotein. |
| Participants | 6 | (*a*) *Cohort study*—Give the eligibility criteria, and the sources and methods of selection of participants. Describe methods of follow-up  *Case-control study*—Give the eligibility criteria, and the sources and methods of case ascertainment and control selection. Give the rationale for the choice of cases and controls  *Cross-sectional study*—Give the eligibility criteria, and the sources and methods of selection of participants | 4-6 | Urgent-start dialysis were defined as ESRD patients who required urgent initiation of dialysis without pre-established functional vascular access or a PD catheter. Patients were grouped according to the dialysis modality (PD and HD). Decisions of when to start dialysis therapy were made by experienced nephrologists on the basis of clinical conditions and laboratory parameters of individual patients. Exclusion criteria included severe respiratory insufficiency, severe acute heart failure, severe hyperkalemia (>6.5 mmol/L), and severe acidosis (serum bicarbonate <12 mmol/L).  In PD patients, all Tenckhoff catheter insertions were performed by experienced nephrologists using the laparotomy method [12] and adhering to one protocol. Low intraperitoneal volume (0.75–1.2 L) was used, which was gradually increased to 2 L per exchange within 2 weeks after catheter insertion in continuous ambulatory peritoneal dialysis, daytime ambulatory peritoneal dialysis, intermittent peritoneal dialysis, or automated peritoneal dialysis (APD). All patients were dialyzed using a glucose-based PD solution (Dianeal; Baxter China, Shanghai, PR China).  All CVCs were inserted in HD patients under local anesthesia by experienced nephrologists following the Seldinger technique [13] in the internal jugular or femoral vein. HD patients were treated by HD for 6–12 h/week (blood flow, 180–200 mL/min; dialysate flow, 500 mL/min; ultrafiltration, 0.4–0.5 L) or by continuous renal replacement therapy (RRT) for 6–24 h/week (replacement fluid flow, 2000 mL/h; dialysate flow, 2000 mL/h; ultrafiltration, 0.4–0.5 L). The choice of dialysis prescription was made on the basis of the clinical status of each individual patient. All CVCs were double-lumen 11.5-F catheters (Mahurkar; Kendall-Tyco Healthcare, Shanghai, China). |
|  |  | (*b*) *Cohort study*—For matched studies, give matching criteria and number of exposed and unexposed  *Case-control study*—For matched studies, give matching criteria and the number of controls per case |  | N/A |
| Variables | 7 | Clearly define all outcomes, exposures, predictors, potential confounders, and effect modifiers. Give diagnostic criteria, if applicable | 5-6 | The primary outcomes of the study were the incidence of dialysis-related complications (infectious and non-infectious complications) and dialysis-related complications requiring catheter re-insertion and bacteremia during the first 30 days after catheter insertion. Dialysis-related complications (episodes, type, intervention strategy, and outcome) and patient outcomes (death, transfer to other centers, or loss to follow-up) were carefully tracked and recorded. |
| Data sources/ measurement | 8* | For each variable of interest, give sources of data and details of methods of assessment (measurement). Describe comparability of assessment methods if there is more than one group | 5-7 | Data recorded at the time of initiating dialysis included age, gender, primary etiology of ESRD, presence of co-morbid diseases (diabetes, hypertension, cardiovascular disease, chronic heart failure [New York Heart Association {NYHA} stage III-IV], cerebrovascular disease, and malignancy, Charlson co-morbidity index [CCI]), use of steroids, and early referral to nephrologists in 6 months. Laboratory parameters were collected at the time of initiating dialysis, including the estimated glomerular filtration rate, serum creatinine, serum urea, serum sodium, serum potassium, pH, serum bicarbonate, brain natriuretic peptide, serum albumin, serum pre-albumin, hemoglobin, serum calcium, serum corrected calcium, serum phosphate, parathyroid hormone, triglycerides, total cholesterol, low-density lipoprotein, and high-density lipoprotein.  Differences between the groups in patient demographics and clinical and laboratory parameters were evaluated by t-test for normally distributed data or Mann­–Whitney non-parametric test for non-normally distributed data. Comparisons of percentages between groups were performed using the chi-square test. Logistic regression analysis was used to determine the factors associated with dialysis-related complications and patient survival rate. Kaplan–Meier curves and log-rank test were used to compare the patient survival rate between groups. |
| Bias | 9 | Describe any efforts to address potential sources of bias |  | Exclusion criteria were used to exclude the most severe ESRD patient in order to address potential sources of bias. |
| Study size | 10 | Explain how the study size was arrived at |  | N/A |

Continued on next page

| Quantitative variables | 11 | Explain how quantitative variables were handled in the analyses. If applicable, describe which groupings were chosen and why | 5-7 | Patients were grouped according to the dialysis modality (PD and HD). All results are expressed as the mean ± standard deviation for normally distributed data and frequencies and percentages for non-normally distributed and categorical data. |
| --- | --- | --- | --- | --- |
| Statistical methods | 12 | (*a*) Describe all statistical methods, including those used to control for confounding | 6-7 | All results are expressed as the mean ± standard deviation for normally distributed data and frequencies and percentages for non-normally distributed and categorical data. Differences between the groups in patient demographics and clinical and laboratory parameters were evaluated by t-test for normally distributed data or Mann­–Whitney non-parametric test for non-normally distributed data. Comparisons of percentages between groups were performed using the chi-square test. Logistic regression analysis was used to determine the factors associated with dialysis-related complications and patient survival rate. Kaplan–Meier curves and log-rank test were used to compare the patient survival rate between groups.  All statistical analyses were performed using SPSS for Windows (version 19.0; SPSS, Inc., Chicago, IL, USA). A p value <0.05 was considered statistically significant. |
|  |  | (*b*) Describe any methods used to examine subgroups and interactions | 7 | Differences between the groups in patient demographics and clinical and laboratory parameters were evaluated by t-test for normally distributed data or Mann­–Whitney non-parametric test for non-normally distributed data. Comparisons of percentages between groups were performed using the chi-square test. Logistic regression analysis was used to determine the factors associated with dialysis-related complications and patient survival rate. Kaplan–Meier curves and log-rank test were used to compare the patient survival rate between groups. |
|  |  | (*c*) Explain how missing data were addressed |  | There was no missing data in our study. |
|  |  | (*d*) *Cohort study*—If applicable, explain how loss to follow-up was addressed  *Case-control study*—If applicable, explain how matching of cases and controls was addressed  *Cross-sectional study*—If applicable, describe analytical methods taking account of sampling strategy |  | N/A |
|  |  | (*e*) Describe any sensitivity analyses |  | N/A |
| Results | | | | |
| Participants | 13* | (a) Report numbers of individuals at each stage of study—eg numbers potentially eligible, examined for eligibility, confirmed eligible, included in the study, completing follow-up, and analysed | 7 | Our study enrolled 178 patients (56.2% male), including 96 (53.9%) PD patients and 82 (46.1%) HD patients. In the PD group, the median break-in period was 4 days. In the HD group, patients started dialysis on the same day or the day after catheter insertion. |
|  |  | (b) Give reasons for non-participation at each stage |  | N/A |
|  |  | (c) Consider use of a flow diagram |  | N/A |
| Descriptive data | 14* | (a) Give characteristics of study participants (eg demographic, clinical, social) and information on exposures and potential confounders | 7-8 | Compared with HD patients, PD patients had more cardiovascular disease, less heart failure, higher levels of serum potassium, hemoglobin, serum albumin, and serum pre-albumin, and lower levels of brain natriuretic peptide. There were no significant differences in terms of gender, age, use of steroids, early referral to a nephrologist in the past 6 months, prevalence of primary renal diseases, prevalence of co-morbidities (diabetes mellitus, hypertension, cerebrovascular diseases, and malignancies), CCI, and other clinical characteristics between the groups (all p >0.05). Tables 1 and 2 show the baseline demographic and clinical characteristics of the study patients. |
|  |  | (b) Indicate number of participants with missing data for each variable of interest |  | None |
|  |  | (c) *Cohort study*—Summarise follow-up time (eg, average and total amount) |  |  |
| Outcome data | 15* | *Cohort study*—Report numbers of outcome events or summary measures over time | 11-15 | During the first 30 days after catheter insertion, 5 PD patients (5.2%) and 20 HD patients (24.4%) developed dialysis-related complications (Table 3). Three PD patients developed non-infectious complications (all malpositioned), but only one required surgical intervention. No patient developed severe or life-threatening complications, such as severe bleeding, leakage, and organ rupture. Two PD patients developed infectious complications (both had peritonitis), and both patients were cured after standard treatment. Among the 20 HD patients who developed dialysis-related complications, all required catheter re-insertion. Eleven HD patients developed non-infectious complications, including bleeding (n = 3), thrombosis (n = 6), and self-removal (n = 2). HD patients had a significantly higher proportion of bacteremia in the first 30 days compared to PD patients (p = 0.011). Among the 11 HD patients who had bacteremia, 9 were related to catheter infections and the other 2 were related to severe pulmonary infections. Causes of bacteremia in the 3 PD patients included pulmonary infection (n = 2) and peritonitis (n = 1).  In each group, the actuarial patient survival at 3 months was 97.9% for PD patients and 98.4% for HD patients. The actuarial patient survival at 1 year was 92.1% for PD patients and 93% for HD patients. Kaplan–Meier curves revealed no significant difference in patient survival between the two groups (Fig. 1). |
|  |  | *Case-control study—*Report numbers in each exposure category, or summary measures of exposure |  |  |
|  |  | *Cross-sectional study—*Report numbers of outcome events or summary measures |  |  |
| Main results | 16 | (*a*) Give unadjusted estimates and, if applicable, confounder-adjusted estimates and their precision (eg, 95% confidence interval). Make clear which confounders were adjusted for and why they were included | 13-15 | After adjustment for demographic and laboratory indicators, urgent-start HD was an independent risk factor in patients with short-term dialysis-related complications (Table 4). Three PD patients (3.1%) and 11 HD patients (13.4%) developed bacteremia within 30 days after catheter insertion. After adjustment for baseline characteristics, low serum potassium and albumin levels were risk factors for patient survival, but the urgent-start dialysis modality was not correlated with patient survival (Table 5). |
|  |  | (*b*) Report category boundaries when continuous variables were categorized |  | N/A |
|  |  | (*c*) If relevant, consider translating estimates of relative risk into absolute risk for a meaningful time period |  | N/A |

Continued on next page

| Other analyses | 17 | Report other analyses done—eg analyses of subgroups and interactions, and sensitivity analyses |  | N/A |
| --- | --- | --- | --- | --- |
| Discussion | | | | |
| Key results | 18 | Summarise key results with reference to study objectives | 15 | This is the first study with a large sample to directly compare short-term complications and patient survival in urgent-start PD and HD patients. Our results suggest that patients who started urgent-start PD experienced a lower risk of short-term dialysis-related complications, but did not correlate with patient survival. Urgent-start HD is an independent risk factor for short-term dialysis-related complications. |
| Limitations | 19 | Discuss limitations of the study, taking into account sources of potential bias or imprecision. Discuss both direction and magnitude of any potential bias | 19 | Our study had several limitations. The study was a single-center, non-matched, retrospective study. The single center nature of the study also limited the generalizability of the results. The dialysis modality was recommended by individual nephrologists based on patient condition and ultimately determined by the patient. Decisions of when to start dialysis therapy were made by an experienced nephrologist on the basis of the clinical status and laboratory parameters of the individual patient. Although all of the nephrologists practicing PD were experienced physicians and would typically make the same decision, individual bias cannot be completely avoided. In addition, urgent-start HD patients were in more critical conditions in our study with lower levels of potassium, hemoglobin, serum albumin, and serum pre-albumin, and higher levels of brain natriuretic peptide. Moreover, logistic regression analysis showed that low serum potassium and albumin levels were risk factors for patient survival, which might result in worse outcomes. Thus, additional research should focus not only on what type of dialysis access should be used, but also on whom. Clearly, prospective randomized controlled trials are needed to definitively demonstrate the optimal dialysis prescription in urgent-start PD patients. |
| Interpretation | 20 | Give a cautious overall interpretation of results considering objectives, limitations, multiplicity of analyses, results from similar studies, and other relevant evidence | 17-19 | Recently, there has been mounting evidence on the feasibility of PD as an alternative to HD as an urgent-start dialysis modality [5–11]. These studies have concluded that PD is equivalent to HD with respect to patient survival, and an additional benefit of PD is fewer short-term dialysis-related complications. In addition, Liu et al. [23] characterized the costs associated with different urgent-start modalities over the first 90 days of treatment from a provider perspective. Liu et al. [23] reported the estimated per patient cost over the first 90 days for urgent-start PD and HD was $16,398 and $19,352, respectively. Thus, urgent-start PD is a cost-saving approach for the initiation of dialysis in patients requiring urgent-start dialysis [23].  Shame et al. [24] reported that after a tight catheter was secured during the insertion, the overall incidence of peri-catheter leakage remained low in the entire study cohort, and the incidence of peri-catheter leakage did not increase despite a shorter break-in period. Povlsen and Ivarsen [5] retrospectively described how acute APD was initiated using a standard prescription for a 12-h overnight APD in the supine position immediately after PD catheter placement and compared short-term outcome measures and dialysis-related complications between urgent-start and planned-start patients. Povlsen and Ivarsen [5] reported that there was no significant difference in the number and type of infectious complications between the two groups despite higher mechanical complications in the acute group compared with the planned group (p <0.05). There was no difference in short-term PD technique survival rates between the two groups (86.7% vs. 90.0%) [5]. A small sample size, prospective, randomized study reported that peritonitis, exit-site infections, catheter-related complications, and other complications were similar between the urgent-start PD and non-urgent-start groups, although the number of minor leaks was higher in the urgent-start PD group [6]. More recently, another small sample size, prospective study showed the safety and feasibility of urgent-start PD in a developing country [7]. Among 35 patients with urgent initiation of PD, peritonitis and mechanical complications occurred in 14.2% and 25.7%, respectively. Technique survival was 85.7% [7]. A previous large size, retrospective study (n = 657) in our center also showed that a break-in period of <1 week might result in a minor increased risk of mechanical complications, but might have no major effect on technique survival in PD patients [25]. In the current study, only five PD patients had dialysis-related complications in the first 30 days after catheter insertion and only one required surgical intervention. None of the complications were severe or life-threatening. Considering our center had extensive experience in PD catheter implantation and management, our findings showed that at least n an experienced center, PD could be an alternative dialysis modality for urgent-start dialysis ESRD patients.  There is limited evidence directly comparing dialysis-related complications between urgent-start PD and HD [8, 9]. Koch et al. [8] showed that unplanned HD patients had a significantly higher probability of bacteremia in the first 183 days compared to PD patients (21.1 vs. 3.0%, p <0.01), whereas the risk for peritonitis was not significantly different in the two groups (1.8% vs. 1.5%, p = 1.000). Consistent with the findings of Koch et al. [8] we found that the incidence of bacteremia was considerably higher in HD patients compared to PD patients.  Lobbedez et al. [9] reported that actuarial patient survival at 1 year was 79% for unplanned HD compared with 83% for unplanned PD. After adjustment of the initial modified CCI, dialysis modality had no impact on patient survival [9]. Koch et al. [8] also reported there was no significant difference in half-year mortality in unplanned PD patients versus unplanned HD patients (30.3% vs. 42.1%, p = 0.19). In the current study, the actuarial patient survival at 3 months and 1 year was 97.9% and 92.1% for PD patients, and 98.4% and 93% for HD patients, respectively. No significant difference in patient survival existed between the two groups. Our findings suggest that urgent-start PD did not have a negative effect on patient survival as an urgent dialysis modality. |
| Generalisability | 21 | Discuss the generalisability (external validity) of the study results | 20 | Our study suggested that peritoneal dialysis is a safe and feasible alternative to hemodialysis for urgent dialysis in ESRD patients at an experienced center. |
| Other information | |  | | |
| Funding | 22 | Give the source of funding and the role of the funders for the present study and, if applicable, for the original study on which the present article is based | 20 | This work was supported by the National Natural Science Foundation of China (grant No. 81370794), the National "Twelfth Five-Year" Plan for Science & Technology (grant No. 2011BAI10B08), and the Research Project of Health Public Welfare Industry in China (grant No. 201502023) . |

*Give information separately for cases and controls in case-control studies and, if applicable, for exposed and unexposed groups in cohort and cross-sectional studies.

**Note:** An Explanation and Elaboration article discusses each checklist item and gives methodological background and published examples of transparent reporting. The STROBE checklist is best used in conjunction with this article (freely available on the Web sites of PLoS Medicine at http://www.plosmedicine.org/, Annals of Internal Medicine at http://www.annals.org/, and Epidemiology at http://www.epidem.com/). Information on the STROBE Initiative is available at www.strobe-statement.org.
